# User-centric design of a 3D search interface for protein-ligand complexes

**DOI:** 10.1007/s10822-024-00563-3

**Published:** 2024-05-30

**Authors:** Konrad Diedrich, Christiane Ehrt, Joel Graef, Martin Poppinga, Norbert Ritter, Matthias Rarey

**Affiliations:** 1 Universität Hamburg, ZBH - Center for Bioinformatics, Albert-Einstein-Ring 8-10, 22761 Hamburg, Germany; 2 Universität Hamburg, Department of Informatics, Vogt-Kölln-Straße 30, 22527 Hamburg, Germany

**Keywords:** Protein-ligand complexes, Molecular interactions, 2D query editor, GeoMine, 3D search engine, PoseEdit, Protein Data Bank, AlphaFold

## Abstract

In this work, we present the frontend of GeoMine and showcase its application, focusing on the new features of its latest version. GeoMine is a search engine for ligand-bound and predicted empty binding sites in the Protein Data Bank. In addition to its basic text-based search functionalities, GeoMine offers a geometric query type for searching binding sites with a specific relative spatial arrangement of chemical features such as heavy atoms and intermolecular interactions. In contrast to a text search that requires simple and easy-to-formulate user input, a 3D input is more complex, and its specification can be challenging for users. GeoMine’s new version aims to address this issue from the graphical user interface perspective by introducing an additional visualization concept and a new query template type. In its latest version, GeoMine extends its query-building capabilities primarily through input formulation in 2D. The 2D editor is fully synchronized with GeoMine’s 3D editor and provides the same functionality. It enables template-free query generation and template-based query selection directly in 2D pose diagrams. In addition, the query generation with the 3D editor now supports predicted empty binding sites for AlphaFold structures as query templates. GeoMine is freely accessible on the Proteins*Plus* web server (https://proteins.plus).

**Supplementary Information:**

The online version contains supplementary material available at 10.1007/s10822-024-00563-3.

## Introduction

A large number of experimentally determined three-dimensional (3D) structures of biological macromolecules are publicly available thanks to the substantial growth of the Protein Data Bank (PDB) [1] and are easily accessible through its web service. This wealth of data is a fundamental scientific resource for understanding macromolecule-ligand interactions and their functional impact. However, to fully exploit this data resource, search engines have to go beyond basic querying on a textual level and enable direct searching of the most central part of the data, the 3D structures themselves. The capability to retrieve all structures with a similar relative spatial arrangement of chemical features like atoms, functional groups, or intermolecular interactions from the PDB can support numerous applications in life science research. For example, searching a query that covers a ligand binding mode within a binding site may result in potential off-target binding sites with similarly interacting ligands, thereby explaining side effects, mining for interaction geometries [2], searching for residue motifs [3], and assisting drug repurposing [4].

In addition to the web service of the PDB itself [5], several tools have been developed that enable specific types of spatial queries for the PDB: CSD-CrossMiner [6], PRDB [7], PROLIX [8], Relibase and Relibase+ [9], PDBeMotif [10], PELIKAN [11], GSP4PDB [12], GeoMine [13, 14], and nAPOLI [15]. In addition, some commercial and unpublished software applications such as Proasis4 [16] and 3decision [17] offer similar search capabilities. Of the published tools, PRDB, PROLIX, Relibase, Relibase+, PDBeMotif, and nAPOLI are no longer available. CSD-CrossMiner and PELIKAN are desktop applications, while GSP4PDB and GeoMine are accessible on the web. GSP4PDB and GeoMine are freely available. CrossMiner and PELIKAN require a commercial or academic license, respectively. The tools differ significantly regarding the supported query content and what regions of the structures in the PDB are searchable. For example, while the PDB web service allows searching the relative spatial arrangement of α- and β-carbon atoms of specific residues in complete protein structures, PELIKAN permits a query that describes a relative spatial arrangement of arbitrary user-specified heavy atoms and intermolecular interactions to screen ligand-bound binding sites defined by a radius of 6.5 Å of the ligand’s heavy atoms. For a comprehensive overview of the query differences and technical aspects of the different tools, like the underlying data storage approach, see [13].

Due to the multidimensional nature of the data and the varying complexity of the supported 3D query, spatial searches are highly challenging, not only from the developer’s point of view but also from the user’s perspective. In contrast to the simple text-based user input of keywords, scalar values, sequences, or even substructures, specifying relative spatial arrangements of chemical features is a complex task. In general, drawing with a graphical editor substantially simplifies query generation, in contrast to defining the query purely textually.

A two-dimensional (2D) or 3D editor provides a more intuitive interface for placing and specifying chemical features and their geometric constraints. Additionally, both editor types already give life scientists a familiar environment for visualizing chemical structures. Generating queries with such an editor can be further simplified by visualizing a structure of interest as a template, in which the user can select the arrangement of chemical features to search for. Nevertheless, an additional textual specification of the 3D query as a manual post-processing step is useful for adapting its chemical and spatial precision to individual needs.

The PDB web service, CSD-CrossMiner, GeoMine, and PELIKAN provide a 3D editor. A template structure for query design can be used in all tools. While a query can be designed anywhere in a loaded 3D representation of a PDB entry using the PDB web service, the query options in CSD-CrossMiner, GeoMine, and PELIKAN are limited to corresponding 3D-visualized binding sites as structural templates. Query generation from scratch is possible with CSD-CrossMiner and GeoMine via the 3D editor and PELIKAN via a textual and tabular representation. PROLIX enables a purely textual approach. All other tools offer a 2D editor for template-free query generation.

Considering a 2D and 3D editor in comparison, both visualization concepts have advantages and disadvantages for generating spatial queries. A 3D environment is a natural choice because it provides precise spatial information. However, the drawback of a 3D editor is that its usage requires practice and time, especially when using a template for query selection. Due to the high amount of visualized structural information, the chemical features of interest might be visually buried inside the structure and must, therefore, be focused on by users by zooming, translating, and rotating the scene extensively. Therefore, query generation can still be challenging, even though a 3D visualization provides all required information.

In contrast, a 2D environment provides only distorted spatial information due to the dimensionality reduction. Furthermore, converting a 3D template structure into a planar representation prevents the visualization of the entire structure due to consequential structural overlaps causing suboptimal 2D layout quality. Therefore, 2D visualization requires a reduction in the amount of visualized structural information. Even though a 2D visualization provides less information than a 3D visualization, it visualizes and highlights only the most relevant chemical information a user might want to search for. Furthermore, 2D visualization offers chemical structure representation as structure diagrams that are very familiar to scientists. A 2D visualization permits an instant overview of the most relevant selectable chemical features, simplifying query generation.

In this article, we will introduce the latest version of GeoMine. First, we will provide a user-focused overview of GeoMine, including its new features: the 2D query editor and the 3D template type based on the artificial intelligence-predicted AlphaFold structures [18] that are retrieved from the corresponding database at https://alphafold.ebi.ac.uk. We will then present the 2D editor in more detail and showcase the application of the new features of the latest tool version, which exploits all the above-mentioned query generation approaches to design a graphical user interface with the highest usability possible for spatial searching within known and predicted binding sites.

## Methods

### Features overview

The key features of the most recent release of GeoMine are summarized in the list below. Subsequently, some of these points are illustrated in detail, including the integration of the new features of GeoMine, the 2D query editor, and the AlphaFold-based 3D template type:

- A graphical user interface that is freely accessible via the Proteins*Plus* [19–21] web server (https://proteins.plus).
- A fast and precise search functionality that enables 3D querying of ligand-bound and predicted empty binding sites of protein or nucleic acid structures in the entire PDB. In the new GeoMine version, binding sites are predicted by DoGSite3 [22]. The binding sites are post-processed by Protoss [23, 24] to calculate the presence and coordinates of polar hydrogen atoms.
- On-the-fly loading of ligand-bound and predicted empty binding sites as query templates created from a PDB structure, an AlphaFold structure, or an uploaded custom structure file in PDB format.
- An interactive and user-friendly query generation process in a 2D and 3D editor that allows synchronized query selection in a ligand-bound or predicted empty 3D template binding site and 2D ligand interaction diagram, respectively, as well as its generation from scratch.
- A large number of selectable chemical features that include all buried and solvent-exposed heavy atoms of all ligands (e.g., solvent molecules, cofactors, small molecules), simple ions like metal ions, and biomolecular residues (amino acid and nucleic acid residues) in a binding site, as well as visualized aromatic ring centers, secondary structure elements, and hydrogen bond, pi-stacking, cation-pi, metal, and ionic interactions. GeoMine allows combining all of these chemical features into a single complex 3D query.
- The placement of hypothetical chemical features in 2D and 3D space for template-free query generation.
- A comprehensive specification of the spatial relationships between chemical features through geometric constraints, which include orientations, distance ranges, and angle ranges.
- A simple verification and arbitrarily precise specification of the query due to its additional representation in tables, which show various properties of the chemical features and geometric constraints that can be adjusted in detail or kept more generic. For example, users can specify whether a selected atom of the polar residue serine matches only serine residues or all polar residues or residues of any type and class.
- The automatic loading of the primary or even all properties of a chemical feature into the query table by chemical feature selection.
- A clear visual correspondence of the query visualized in the 2D and 3D editors and the tables achieved by synchronized mouse-over highlighting and individual coloring for the chemical features and geometric constraints of the query.
- A user-specified ulterior restriction of the search in the PDB by an optional list of PDB identifiers and by the inclusion or exclusion of results based on 53 additional textual and numerical filter criteria, such as the source organism, the protein class, or the root-mean-square deviation (RMSD) between the match and query points.
- An iterative search process of query editing and subsequent searching in already-detected results enabled by a refinement functionality and results history.
- The download and upload of a GeoMine query in JavaScript Object Notation (JSON) file format for sharing, archiving, reusing, and later editing.
- A comprehensive presentation and comparative analysis of the resulting binding sites by a table with information about the 150 best results and the visualization of these in the 3D editor together with the 3D template binding site as superimpositions of the template and matching binding sites with various 3D visualization options. The ranking and superimpositions of results are based on the RMSD between the chemical point features of the query (atoms, aromatic ring centers, secondary structure elements) and the corresponding ones of the matches.
- The download of the table content in JSON or comma-separated values (CSV) format, of the superposed binding sites of the 150 best results in PDB format, and of a file that contains the statistics for all matches.

### New features integration

**Fig. 1:**
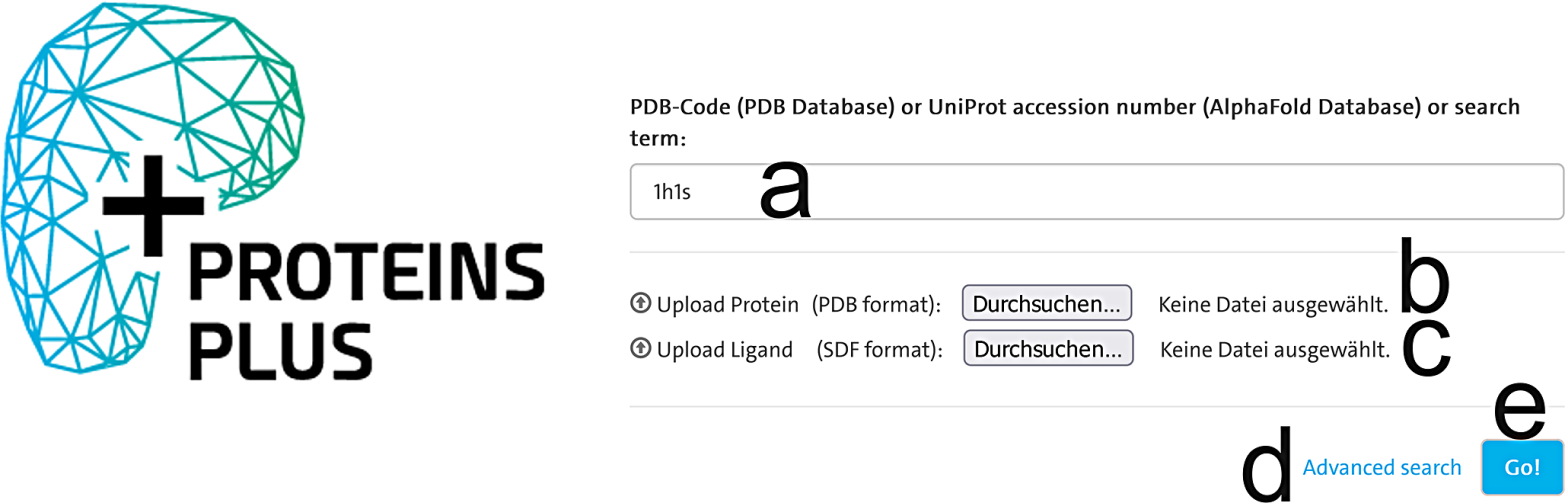
Excerpt of the landing page of Proteins*Plus. ***a** Text field for the specification of a Protein Data Bank or AlphaFold structure as input. **b** Upload button for a PDB file with a custom input structure. **c** Upload button for an SDF file with additional ligands. **d** Link to the advanced search functionality. **e** Button for the confirmation of the input structure

**Fig. 2:**
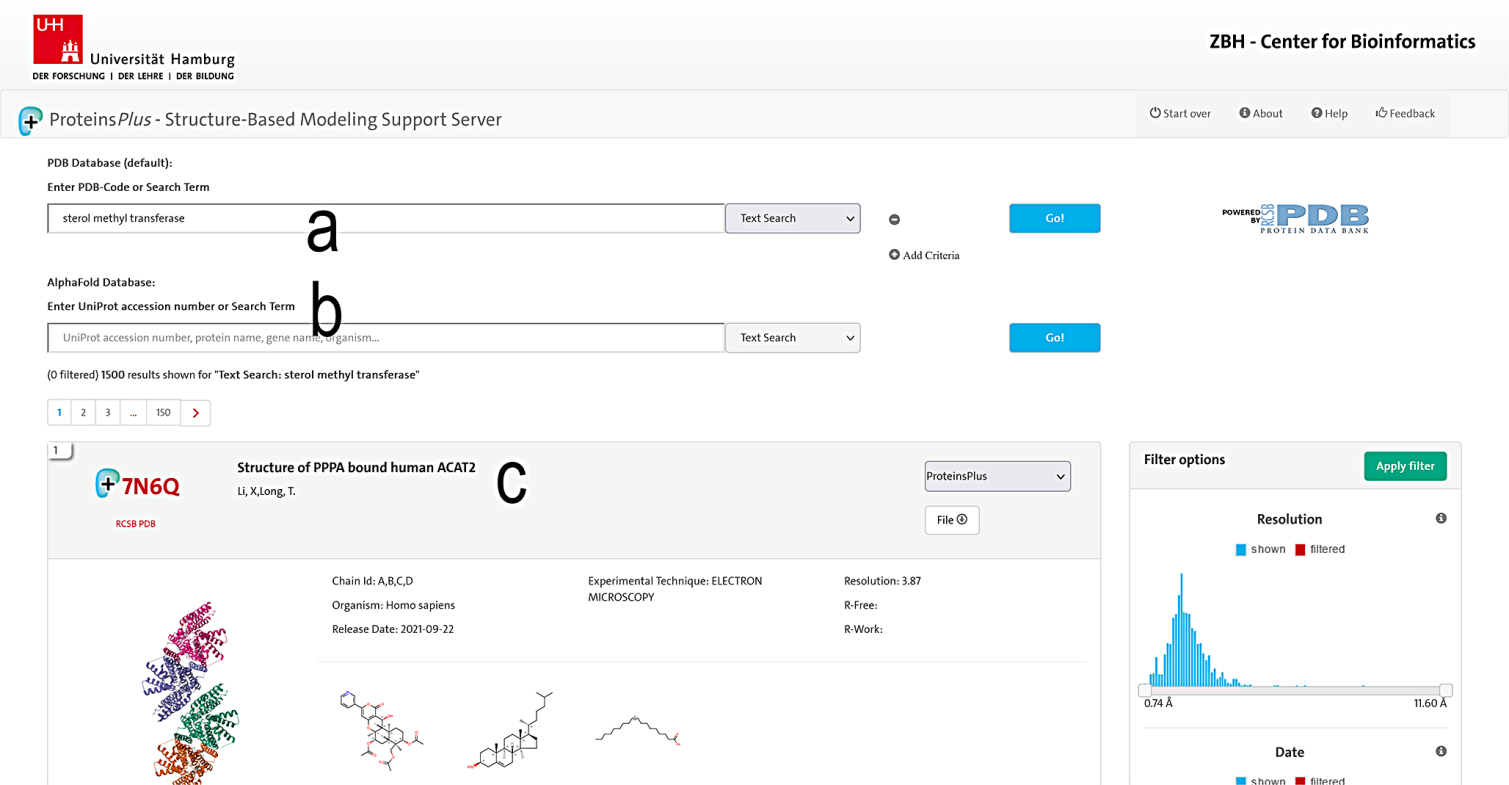
Advanced search functionality of Proteins*Plus*. **a** Text field for the keyword-based querying of the Protein Data Bank. **b** Text field for the keyword-based querying of the AlphaFold database. **c** List of search results

The user can specify a template structure for query generation on the Proteins*Plus* landing page (Fig. 1) in several ways. Besides the specification of a Protein Data Bank structure by its 4-letter identifier (Fig. 1a) or a custom structure by a file in PDB format (Fig. 1b), the user can now directly access predicted structures in the AlphaFold database through their UniProt accession numbers. Additional ligands can be uploaded in Structural Data File (SDF) format (Fig. 1c) for the specified template structure. The linked advanced search functionality (Fig. 1d) allows the user to query the Protein Data Bank (Fig. 2a) and AlphaFold database (Fig. 2b) by keywords to search for potential input structures (Fig. 2c).

**Fig. 3:**
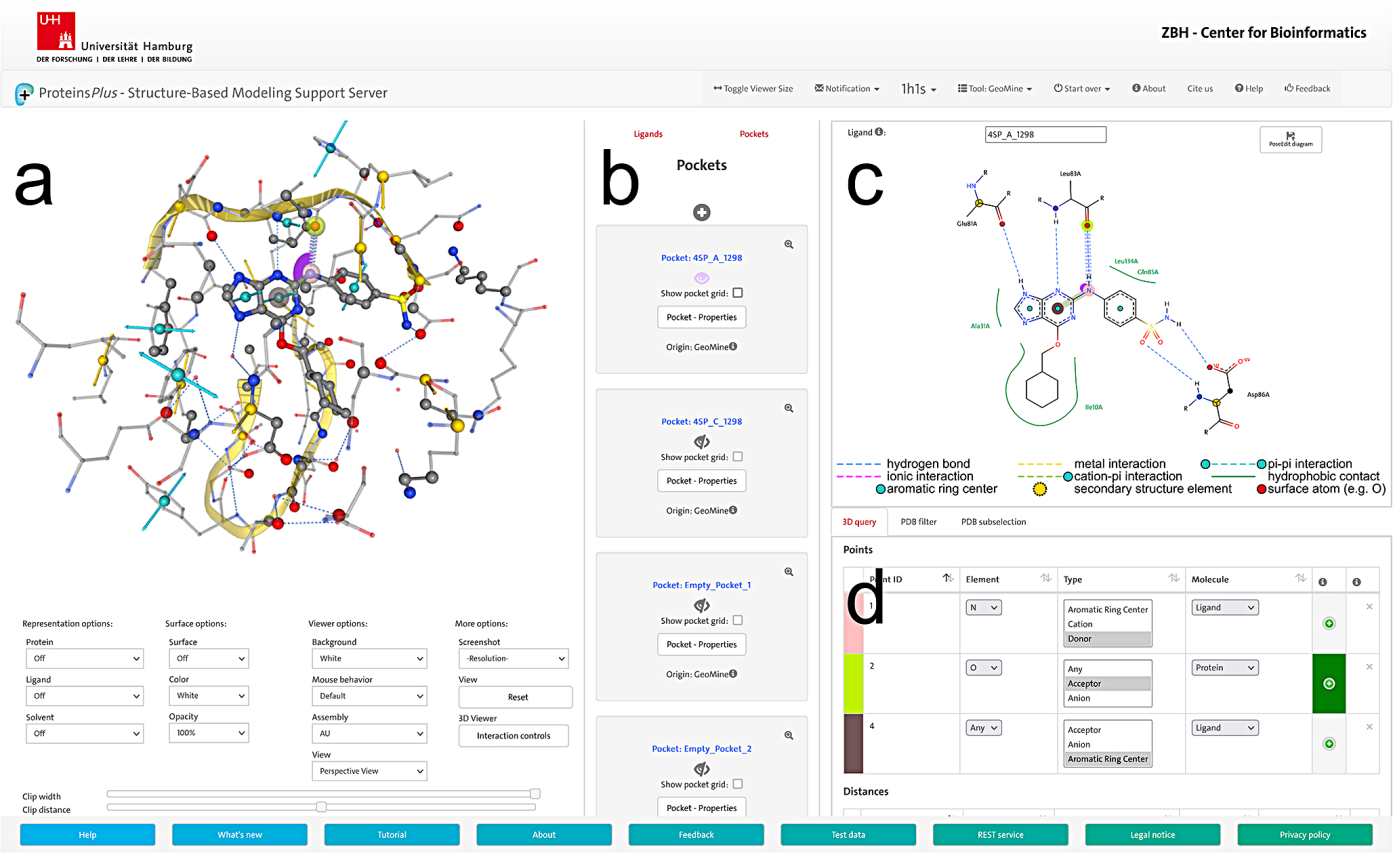
Main page of Proteins*Plus* showing the main components of the graphical user interface of GeoMine and a query. **a** 3D viewer showing a ligand-bound 3D binding site and the query. **b** List of ligand-bound and predicted binding sites of the input structure for visualization in the 3D viewer. A toggleable list of small molecules and ions of the input structure for visualizing the corresponding 2D interaction diagrams in the 2D viewer can be shown by clicking on “Ligands”. **c** 2D viewer showing a 2D ligand interaction diagram and the query. **d** Scrollable section for the query representation by tables, with separate tables for query points, distances, angles, and interactions. In the figure, the focus is on the points table

After input confirmation on the Proteins*Plus* landing page (Fig. 1e), the user is forwarded to the Proteins*Plus* main page (Fig. 3), which consists of three scrollable sections. The user can select GeoMine from the tool list in the right section to access the tool-specific graphical user interface components, including the new 2D query interface. The central section provides two scrollable lists: the *Pockets* and *Ligands* lists (Fig. 3b). The *Ligands* list contains information about all ions and small molecules of the input structure. The *Pockets* list provides information about on-the-fly calculated ligand-bound and DoGSite3-predicted empty binding sites. Ligand-bound binding sites are predicted with a so-called “ligand-bias” option, i.e., the solvent grids are biased by the buried fragments of the ligand to enforce these parts to be included in the predicted sites (ligand-biased predicted sites, see [22] for details). A ligand might not be contained in any DoGSite3-predicted binding site, i.e., less than 20% of its heavy atoms lie in the pocket. In this case, a ligand radius-based binding site is created instead, including the ligand and all residues, other small molecules, and simple ions within a radius of 6.5 Å of the ligand’s heavy atoms. In the case of AlphaFold-based input, only predicted empty binding sites are available, as those structures do not contain ligands. A 2D ligand interaction diagram created with PoseEdit [25] and PoseView [26–28] as a template for query selection can be loaded for a user-specified ligand from the *Ligands* list into the 2D editor on the right (Fig. 3c). The corresponding ligand-bound 3D binding site from the *Pockets* list is then automatically visualized in the 3D editor on the left (Fig. 3a).

**Fig. 4:**
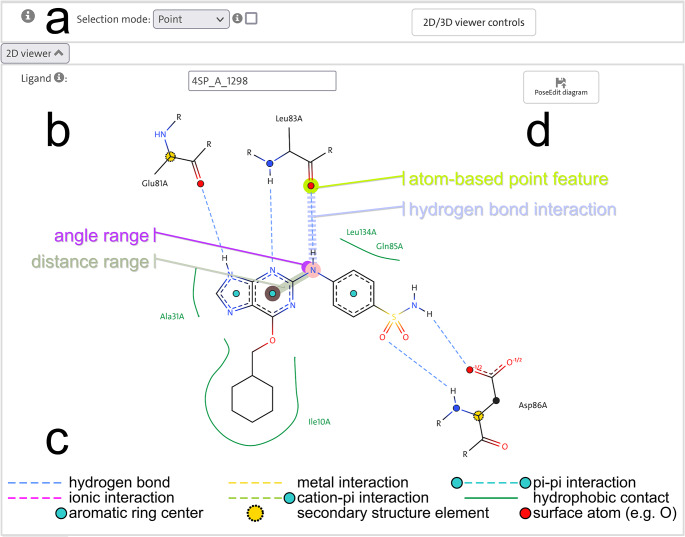
Excerpt of the Proteins*Plus* main page, showing the 2D editor of GeoMine with a 2D ligand interaction diagram and all possible query components. **a** List of modes for query generation in 2D and 3D. **b** Drawing area displaying a 2D diagram of the inhibitor with the internal Proteins*Plus* ID 4SP_A_1298 interacting with a cyclin-dependent kinase (PDB code: 1H1S) [29]. **c** Legend illustrating chemical features. **d** Button for uploading diagram files

Figure 4 provides a detailed view of the 2D ligand interaction diagram content, the 2D editor functionality, and the supported components of the query. A 2D ligand interaction diagram (Fig. 4b) shows an excerpt of the corresponding ligand-bound 3D binding site. The selectable chemical feature types are the same as in the 3D binding sites, but the visualized content is restricted to a specific ligand and directly interacting metals and macromolecular residues. Hydrophobic contacts with residues are not visualized in atomic detail but are indicated by green splines labeled by the corresponding residue identifiers. It is not possible to generate 2D diagrams for predicted empty binding sites. The substantial quantity of solvent-exposed residues in such a binding site cannot be effectively limited, as it is difficult to automatically specify which residues might be more important than others for query selection. A 2D diagram that displays all binding site residues is overly crowded and does not provide any chemical reference point to the user on what to select, rendering the query formulation in 2D space an ineffective alternative. In contrast, a query selection in a predicted empty 3D binding site is more feasible since residues are distinguishable on a spatial level. For example, a user might want to select specific solvent-exposed atoms of nearby residues surrounding a distinct subsection of the binding site. However, for ligand-bound 3D binding sites, it is possible to highlight the ligand, its interaction partners, and the intermolecular interactions in a 2D diagram. This focus increases the clarity of 2D diagrams while providing chemical information useful for query selection even without spatial information.

In addition to the input specification via the *Ligands* list, users can upload a diagram file in JSON format (Fig. 4d). This upload functionality is particularly useful when users want to improve the automatically generated 2D layout for query selection. With the 2D diagram editing tool PoseEdit, which is also accessible on Proteins*Plus*, the user can load and visualize the same 2D diagram to manually rearrange its content for resolving graphical issues like overlapping residues or intersecting intermolecular interactions. The optimized 2D diagram can be downloaded from PoseEdit as a JSON file and can then be uploaded into the 2D editor of GeoMine.

The 2D editor has the same query-building functionality as the 3D editor. Furthermore, the 2D editor is synchronized with the 3D editor and the query tables regarding query generation, visualization, mouse-over highlighting, and coloring. This synchronization allows the simultaneous usage of all query input types in a complementary manner. The query consists of chemical features and geometric constraints that can be added without a template or selected in a template via several user modes (Fig. 4a). A legend below the 2D drawing area (Fig. 4c) explains the precalculated chemical features.

In the *Point* mode, the user can select so-called points, i.e., heavy atoms, aromatic ring centers, and secondary structure elements, represented by α-carbon atoms of central or terminal protein residues in helices and strands. Solvent-exposed heavy atoms are highlighted by big colored spheres. Like in the 3D editor, hypothetical points can be placed and moved in 2D space. They are automatically placed in the center of the ligand-bound 3D binding site that corresponds to the 2D ligand interaction diagram. The relative position of a hypothetical point can be adjusted via the 3D editor and by distance ranges. Intermolecular interactions are visualized by colored dashed lines and can be selected in the *Interaction* mode. It is also possible to specify a hypothetical intermolecular interaction between two points in that mode. Any two points can be connected by a distance range in the *Distance* mode. Lastly, angle ranges can be placed between connected distance pairs and interactions in the *Angle* mode.

The corresponding tables in the scrollable section below the 2D editor list defined points, distances, interactions, and angles (Fig. 3d). The tables allow further verification and modification of their properties, for example, the residue an atom belongs to or the tolerance value of a distance range. The user can specify that all properties of a selected chemical feature are automatically recognized and set in its corresponding table entry after selection by enabling the checkbox next to the list of modes. Otherwise, only its main properties are set automatically, i.e., the element for atoms and the molecule type for atoms, aromatic ring centers, and secondary structure elements. For a screen recording video demonstrating how to apply the user modes, see Online Resource 1. A 2D diagram of the inhibitor with the internal Proteins*Plus* ID 4SP_A_1298 interacting with a cyclin-dependent kinase (PDB code: 1H1S) is shown in the video to exemplify query generation with the 2D editor.

### Technical implementation details

The graphical user interface is primarily implemented with HTML, Vanilla JavaScript, and the Bootstrap 3 library (https://getbootstrap.com). Several JavaScript libraries were used to integrate specific frontend components. The 3D viewer uses the NGL library [30, 31] (https://nglviewer.org). The query tables employ the DataTables library (https://datatables.net). The 2D editor is based on the InteractionDrawer JavaScript library (https://github.com/rareylab/InteractionDrawer), which draws interactive 2D ligand interaction diagrams in Scalable Vector Graphics (SVG) format. The web server’s backend is implemented using the Ruby on Rails framework (https://rubyonrails.org) and a MySQL database (https://www.mysql.com).

GeoMine’s searches are performed on a server using a PostgreSQL (https://www.postgresql.org) database, 200 GB of main memory, up to 30 cores of a 2x Intel Xeon Gold 6248 processor (2.5 GHz), and a Dell 1.6 TB NVMe HHHL AIC PM1725b solid-state drive with an XFS file system.

## Application

### Binding site function prediction and off-target analyses for methyltransferases in *Leishmania*

In our case study, we want to illustrate how GeoMine can be used to analyze AlphaFold models and assist in suggesting ligands and their binding modes for a predicted protein structure of interest. The resulting complexes can subsequently be used to assess the uniqueness of the 3D arrangement of ligand-interacting binding site atoms using 2D query design. Here, we want to focus on neglected tropical diseases threatening millions worldwide [32]. Their treatment is restricted to a few medications that often harbor severe side effects [33]. Causative agents for these diseases are, among others, parasites of the genus *Leishmania*. The search for potential therapeutic agents became the focus of academic infection research, which identified several pharmaceutically promising targets [34]. Understanding their structure and function is crucial for future early-phase drug discovery and development.

The protein of interest in this case study is an enzyme called sterol 24-C methyltransferase (SMT) in *Leishmania* species. The enzyme uses S-adenosyl methionine (SAM) as a cosubstrate and catalyzes the C-C bond formation between a methyl group and the C24 of zymosterol to form ergosterol [35], the major sterol component of these parasites. Several substrate-based inhibitors of the enzyme from *Leishmania amazonensis* are known [36] and a recent computational study aimed to design novel inhibitors [37]. The authors focused their analyses on the zymosterol binding site of the protein to find novel inhibitors. In contrast, we wondered whether the SAM-binding site might provide a suitable starting point for structure-based design. Due to the unavailability of experimental structures, we used the AlphaFold model of the enzyme from *L. donovani* (UniProt Accession Q6RW42).

**Fig. 5:**
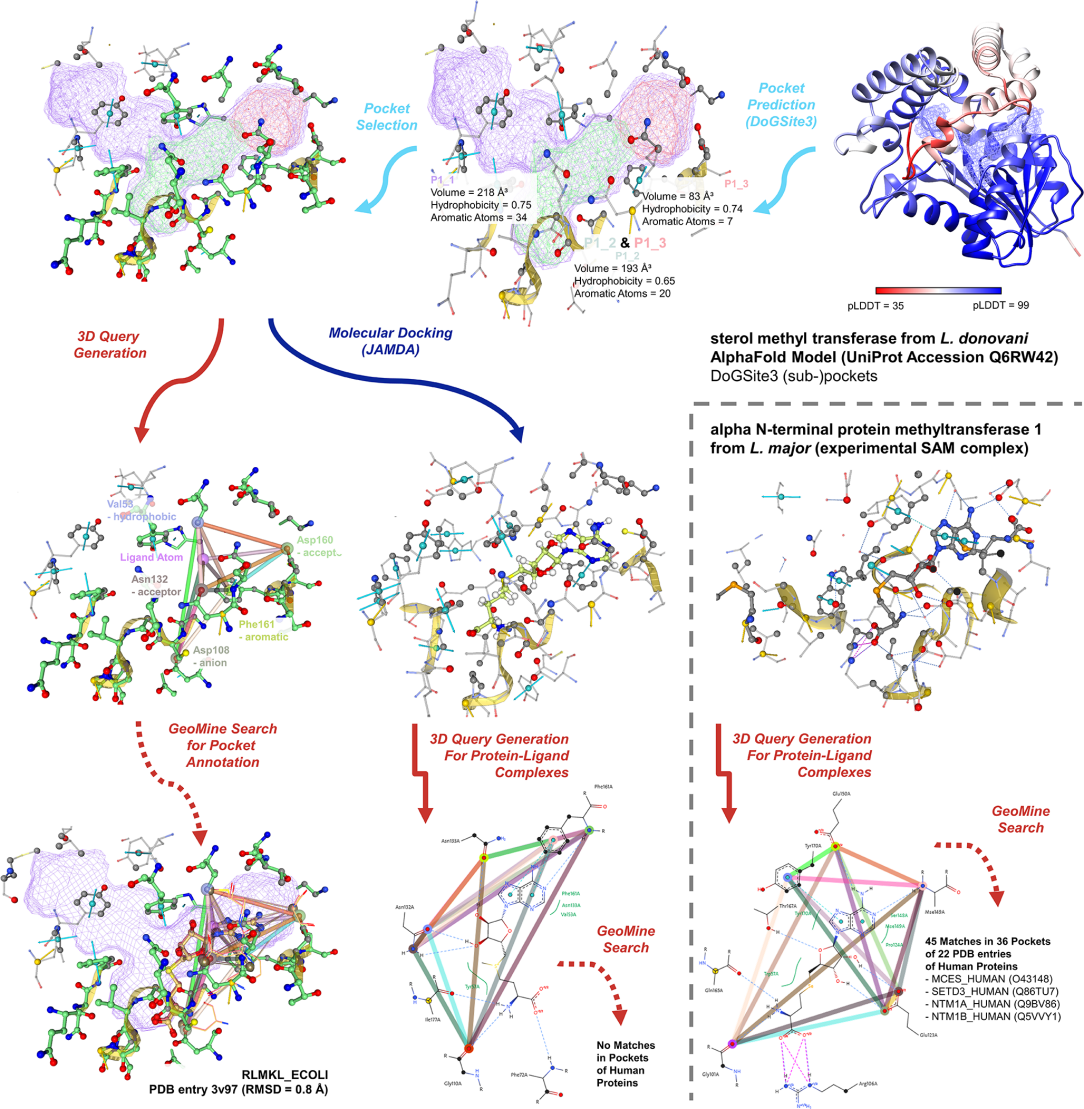
Ligand annotation and off-target prediction for binding sites of *Leishmania donovani* sterol C-24 methyltransferase. The explored workflow involves binding site prediction by DoGSite3 (light blue), molecular docking with JAMDA (dark blue), and the search for related binding sites of SAM, SAH, or SFG in the complete PDB for binding pose comparison and a subset of human structures for searching potential off-targets using GeoMine (predicted site 3D query generation and complex 2D query generation, red). The query for the initial GeoMine search with a predicted site was based on manually chosen solvent-exposed atoms. The pharmacophoric properties of the selected atoms were used as query points (hydrogen bond acceptors and donors, aromatic centers, and hydrophobic atoms). For the prediction of related sites in human protein structures, i.e., pockets with similar interaction patterns to the cosubstrate SAM, with the 2D editor, the point features of oxygen and nitrogen atoms were set as solvent-accessible hydrogen bond donors and acceptors, respectively, if they are involved in hydrogen bonds with the ligand. Aromatic centers were modeled if they undergo pi-pi interactions with the ligand. Independent of the query type, all modeled points connected by distances below 14 Å were annotated by distance restraints with tolerances of 1 Å. Note that only point-point distance restraints up to 15 Å can be defined in the frontend

Upon loading the structure on Proteins*Plus* by entering its UniProt Accession, we can see the ligand-free structure of the protein. In the Pockets tab, we see two pockets predicted by DoGSite3 for the structure. The first is very large, with a volume of 587 Å³ (P1), while the second is much smaller and mainly occupied by charged residues (P2). We conclude that the first pocket might be the active site responsible for SAM and zymosterol binding. DoGSite3 detects three subpockets in this binding site: a large one with many aromatic atoms and a hydrophobicity ratio of 0.76, which is flanked by residues with low pLDDT scores (P1_1), and two smaller ones with lower predicted hydrophobicity and high pLDDT scores (P1_2 and P1_3, Fig. 5). Therefore, we hypothesized that the smaller subpockets might be the site binding to SAM and rely on these subpockets with an overall higher predicted accuracy in terms of pLDDT.

We performed a molecular docking of SAM with JAMDA [38, 39] into these combined subpockets. However, we obtained highly diverse potential poses partially extending to the P1_1 subpocket. Due to structural uncertainties of the structural model representing a considerable challenge for molecular docking [40], the best-scored pose might not correspond to the native binding mode. To find the most probable of the predicted binding poses, we built a GeoMine model based on flanking solvent-exposed binding site residues (Fig. 5) and a point indicating the position of the ligand and screened for similar binding sites in complex with SAM, its enzymatic product S-adenosyl homocysteine (SAH), or their analog sinefungin (SFG). The corresponding query file in JSON format is available in the Supplementary Information (Online Resource 2). The search finished in 21 s. Intriguingly, we found only one protein ligand-complex with SAH that did not clash considerably with the query protein residues: the SAM-binding pocket of ribosomal RNA large subunit methyltransferase K/L from *Escherichia coli* (strain K12, PDB code 3v97). The JAMDA pose on rank 6 is similar to the one in the RNA methyltransferase aligned with GeoMine and might provide a reliable binding hypothesis.

One well-known issue of targeting SAM-binding sites is the comparatively high risk of off-target effects and corresponding toxicity when addressing similar conserved interaction patterns in related enzymes [41]. Although we find highly specialized classes of SAM-binding enzymes in nature [42], a close examination of the interaction pattern similarities might help to identify selectivity-mediating site properties and prevent the design of non-selective inhibitors. Therefore, we further explored the unique features of the binding site. We saved the JAMDA pose on rank 6 and uploaded it as complex to Proteins*Plus*. The corresponding PDB file is available in the Supplementary Information (Online Resource 3). Next, we used the 2D query feature of GeoMine to model residue atoms potentially interacting with SAM-related compounds. As the binding site is highly buried and the number of interactions is high, it is more convenient to use the 2D representation of the interacting partners in this case. We modeled the pharmacophoric properties of all interacting atoms except for the residues interacting with the carboxylic group of the methionyl moiety and backbone atom of Ile177, as those atoms are far apart from the adenosyl moiety. The resulting query was used to screen for related binding sites of human protein structures in the PDB. The corresponding query file in JSON format is available in the Supplementary Information (Online Resource 4). The search took 19 s. Intriguingly, we could not identify similarities in the SAM binding mode predicted for SMT to the one observed for any human enzymes of known structure in complex with SAM, SAH, or SFG, indicating a unique interaction pattern in this protein.

**Fig. 6:**
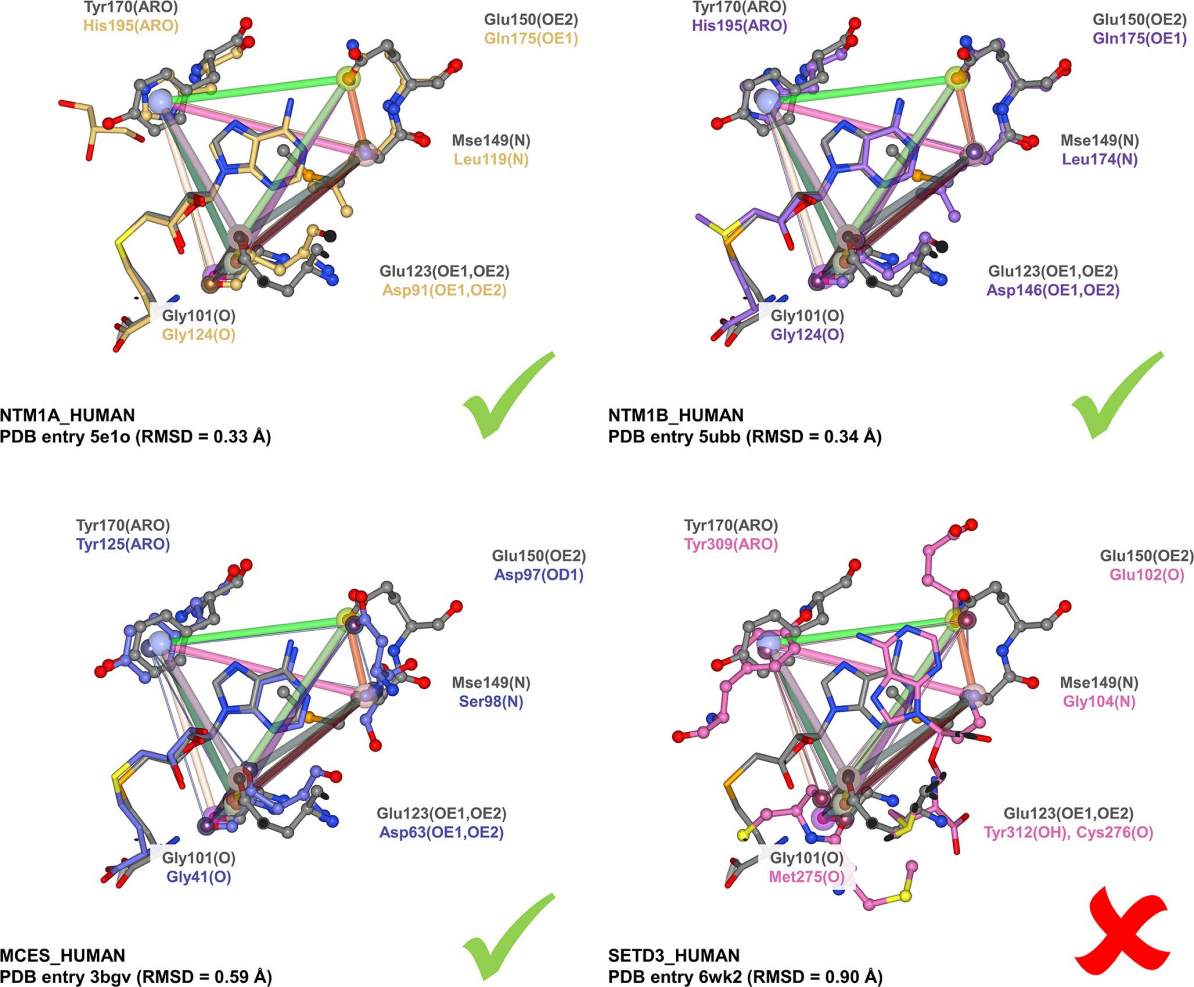
Off-Target Prediction for the SAM-binding site of alpha N-terminal protein methyltransferase 1 from *L. major*. The presented aligned matches are based on the query in Fig. 5 for the binding site of PDB entry 1xtp. The aligned matches of N-terminal Xaa-Pro-Lys N-methyltransferase 1 (PDB entry 5e1o), N-terminal Xaa-Pro-Lys N-methyltransferase 2 (PDB entry 5ubb), and mRNA cap guanine-N7 methyltransferase (PDB entry 3bgv) show striking correspondences in the spatial arrangement of interacting atoms and residue types. They should be considered potential off-targets. In contrast, the match with actin-histidine N-methyltransferase does not lead to a convincing alignment. Additionally, the ligand clashes with binding site residues of the query, indicating a different interaction pattern with SAM

To compare this finding to the results of similar approaches with other SAM-binding enzymes from *L. donovani*, we used another SAM binding site of the enzyme alpha N-terminal protein methyltransferase 1 (UniProt accession number A0A3S7X350). A SIENA [43] search in the PDB revealed a highly related SAM-bound structure of the enzyme of *L. major* (PDB entry 1xtp by the Structural Genomics of Pathogenic Protozoa Consortium). The tool searches for closely related binding sites of other proteins based on perfect k-mer sequence matches in an indexed database of the PDB. As the residues of both active sites overlap nearly perfectly and there are no mutations or gaps in a 5 Å environment, we used a similar GeoMine search strategy to find potentially related sites for this target. As described previously for SMT, we modeled all interacting residue atoms and their distances, omitting the atoms interacting with the carboxylic group of the methionyl moiety and the backbone oxygen atom of Gln165. We omitted the oxygen atom of Thr167 as the ether might represent a comparably weak acceptor. The corresponding query file in JSON format is available in the Supplementary Information (Online Resource 5). The search was performed in 31 s. In contrast to our findings for SMT, we identify several binding sites in human enzymes that are structurally highly related (Fig. 6). The low RMSD values of the matched points indicate a high validity of the hits in terms of matching interacting atoms. A visual inspection of the matches highlights that mainly human N-terminal Xaa-Pro-Lys N-methyltransferase 1, N-terminal Xaa-Pro-Lys N-methyltransferase 2, and mRNA cap guanine-N7 methyltransferase should be considered potential off-targets of compounds addressing similar interacting residues of the SAM site. The match with actin-histidine N-methyltransferase does not lead to a convincing ligand alignment, indicating that the site of this enzyme is different regarding the atoms interacting with SAM. This result suggests that selectively inhibiting this binding site might be more challenging than addressing the one for SMT with an SAM-competitive inhibitor.

In summary, this study illustrates how GeoMine can support the analysis of protein structures concerning ligand binding in just one of the numerous imaginable workflows. Using DoGSite3, putative sites, e.g., from predicted protein structures, can be used as starting points. The fully integrated 2D and 3D query design options paired with the efficient database search capabilities of GeoMine enable on-the-fly structural investigations exploiting data from hundreds of thousands of protein structures. The new functionalities provide easy access to binding site function prediction and automated searches for potential off-targets.

## Conclusion

In this article, we present features and exemplary applications of the new version of GeoMine, a search engine for 3D searching in ligand-bound and predicted empty protein binding sites. Exploiting the full capabilities of such a search engine is a considerable challenge from the user’s perspective due to the complexity of 3D molecular arrangements on the atomistic level being part of the query. In related tools, the 3D query formulation is based on either 2D, 3D, or text input. Each of these input types has advantages and disadvantages.

The new version of GeoMine seamlessly integrates all three input types to maximize the usability of the complex 3D query-building process. The newly implemented 2D editor enables a simplified template-free query generation and template-based query selection for ligand-bound binding sites. The 2D templates make optimal use of the editor’s limited 2D space by highlighting only those chemical aspects of the binding site that are most relevant to the ligand’s interaction with a macromolecule and, therefore, particularly interesting to search for. The 2D editor is instantaneously synchronized with the 3D editor and the textual query representation in tables, enabling a synergistic query generation process complemented by all three input types. A seamless integration of the PoseEdit features into GeoMine might further improve the usability of the 2D interface. Finally, predicted empty binding sites of artificial intelligence-based protein structure models can now be used as 3D templates in the 3D editor, giving the user a new starting point to tailor queries of interest to elucidate potential ligands.

The search engine’s extended graphical user interface will support life scientists in effortlessly generating structural 3D queries on the PDB for the functional analysis of macromolecule-ligand interfaces.

## Electronic supplementary material

Below is the link to the electronic supplementary material.

Supplementary Material 1

Supplementary Material 2

Supplementary Material 3

Supplementary Material 4

Supplementary Material 5

## Data Availability

All data generated or analyzed during this study are included in this published article and its supplementary information files.

## Declarations

### Software availability

The GeoMine application is available at https://proteins.plus. The code of the InteractionDrawer library used for the drawing of interactive 2D diagrams is available at https://github.com/rareylab/InteractionDrawer.

## Abbreviations

3D: Three-dimensional
PDB: Protein Data Bank
2D: Two-dimensional
RMSD: Root-mean-square deviation
JSON: JavaScript Object Notation
CSV: Comma-separated values
SDF: Structural Data File
SVG: Scalable Vector Graphics
SMT: Sterol 24-C methyltransferase
SAM: S-adenosyl methionine
SAH: S-adenosyl homocysteine
SFG: Sinefungin
